# Spike Train Similarity Space (SSIMS) Method Detects Effects of Obstacle Proximity and Experience on Temporal Patterning of Bat Biosonar

**DOI:** 10.3389/fnbeh.2018.00013

**Published:** 2018-02-08

**Authors:** Alyssa W. Accomando, Carlos E. Vargas-Irwin, James A. Simmons

**Affiliations:** ^1^Department of Neuroscience, Division of Biology and Medicine, Brown University, Providence, RI, United States; ^2^National Marine Mammal Foundation, San Diego, CA, United States

**Keywords:** echolocation, biosonar, big brown bat, temporal patterning, memory, clutter, sonar sound groups, spike train similarity space (SSIMS)

## Abstract

Bats emit biosonar pulses in complex temporal patterns that change to accommodate dynamic surroundings. Efforts to quantify these patterns have included analyses of inter-pulse intervals, sonar sound groups, and changes in individual signal parameters such as duration or frequency. Here, the similarity in temporal structure between trains of biosonar pulses is assessed. The spike train similarity space (SSIMS) algorithm, originally designed for neural activity pattern analysis, was applied to determine which features of the environment influence temporal patterning of pulses emitted by flying big brown bats, *Eptesicus fuscus*. In these laboratory experiments, bats flew down a flight corridor through an obstacle array. The corridor varied in width (100, 70, or 40 cm) and shape (straight or curved). Using a relational point-process framework, SSIMS was able to discriminate between echolocation call sequences recorded from flights in each of the corridor widths. SSIMS was also able to tell the difference between pulse trains recorded during flights where corridor shape through the obstacle array matched the previous trials (fixed, or expected) as opposed to those recorded from flights with randomized corridor shape (variable, or unexpected), but only for the flight path shape in which the bats had previous training. The results show that experience influences the temporal patterns with which bats emit their echolocation calls. It is demonstrated that obstacle proximity to the bat affects call patterns more dramatically than flight path shape.

## Introduction

**FIGURE 1:**
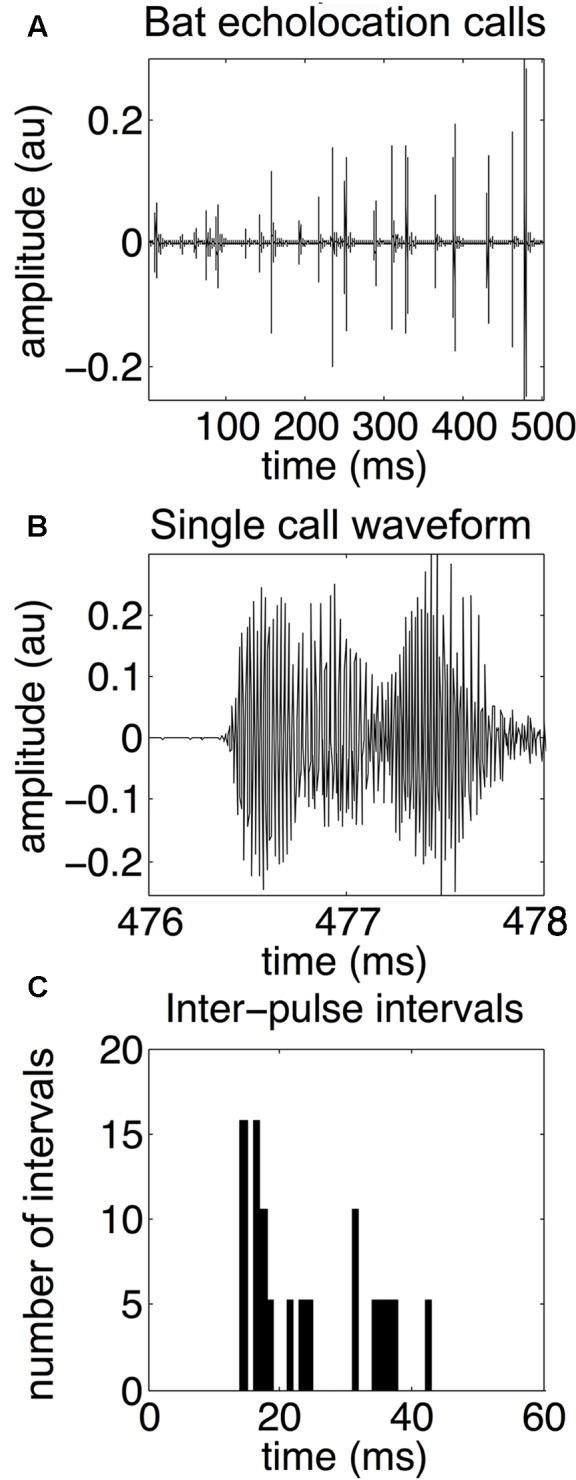
Bat echolocation calls. Bat echolocation calls are shown over a 500-millisecond time period **(A)**, a single call waveform **(B)**, and a histogram showing the distribution of inter-pulse intervals, or IPIs **(C)**. In this example, microphone recordings of the bat’s echolocation signals are sampled at 192 kHz. The call shown in the middle panel is from the same recording as the top panel over the 476–478 ms time frame. Note that both “call” and “pulse” refer to the same type of echolocation vocalization. Notice the bimodal distribution of IPIs. Long IPIs occur between sonar sound groups and are 30–50 ms long, while shorter IPIs occur within a sonar sound group, and are approximately 15–25 ms long. Adapted with permission from Accomando et al. (2017).

An echolocating bat emits high frequency biosonar pulses and listens for returning echoes. The bat uses echo delay and spectra of reflections produced by objects in the environment as cues to construct perceptual images of its surroundings (Moss and Surlykke, 2010; Simmons, 2012). In response to different environmental challenges, the big brown bat (*Eptesicus fuscus*) dynamically adapts its biosonar emissions (Griffin, 1958; Griffin and Grinnell, 1958; Simmons et al., 1998; Surlykke and Moss, 2000). The most well-studied example of biosonar adaptability is prey capture behavior. Bats decrease call duration and intensity, and increase the bandwidth of their broadcasts as they approach prey (Griffin et al., 1960; Kalko and Schnitzler, 1998; Moss and Surlykke, 2001, 2010). Bats can adapt the duration, intensity, and frequency content of their echolocation calls to suit their particular needs at any moment. For example, bats shift their call frequency away from that of conspecifics foraging nearby – a strategy for sonar jamming avoidance (Bates et al., 2008). However, the most important – and most flexible – call parameter is the timing between calls. Whenever possible, bats emit an echolocation call and then wait for the echoes to return before emitting the next sound. However, this is not always possible. Bats routinely forage in cluttered acoustic environments where multiple objects might obstruct the flight path. In these cases, bats must quickly emit more calls to update their perceptual image of the scene rapidly enough to avoid colliding with obstacles. See **Figure 1** for an example echolocation call sequence, waveform of a single call, and the distributon of IPIs.

To effectively navigate, hunt insect prey, and avoid collision hazards in a cluttered environment, bats emit trains of pulses at a higher rate than in an open environment (Surlykke and Moss, 2000; Falk et al., 2014). The time duration between individual pulses in a train is called the inter-pulse interval (IPI). The big brown bat does not uniformly increase or decrease IPIs between pulses. Instead, pulses are emitted in groups, with shorter time intervals (∼20 ms) between pulses in the same group and longer intervals (∼40 ms or longer) between groups (Moss et al., 2006; Petrites et al., 2009; Kothari et al., 2014). Sonar sound groups (also called “strobe groups”) are thought to be an adaptive strategy for bats echolocating in complex environments because an increase in the difficulty of the task or the proximity and distribution of objects in the surroundings is associated with increased sound group emission (Surlykke and Moss, 2000; Petrites et al., 2009; Kothari et al., 2014; Warnecke et al., 2015). The bat’s familiarity with the scene may also impact sonar sound patterning (Barchi et al., 2013; Kothari et al., 2014; Knowles et al., 2015; Hom et al., 2016). It is unknown whether expectation can influence biosonar pulse timing, partly because it is difficult to quantify global temporal patterns of sonar sound sequences across different experimental paradigms, and because different laboratory groups use different metrics for defining sonar sound groups. Temporal patterns in bat echolocation sounds are partially responsible for their ability to fly in cluttered environments with ease. Specifically, sonar sound groups may be a strategy for avoiding pulse-echo ambiguity – a classic sonar engineering problem where the receiver cannot determine which echo resulted from which pulse when pulses are repeated rapidly (Stimson, 1998; Denny, 2007; Wheeler et al., 2016).

**FIGURE 2:**
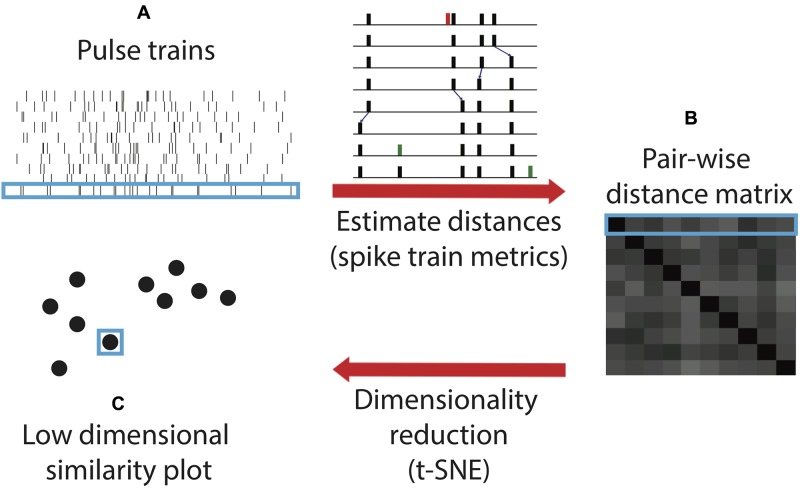
Spike train similarity (SSIMS) algorithm processing steps. The input of the algorithm is a series of pulse trains **(A)**. A matrix pair-wise distances between the pulse trains **(B)** are calculated using spike train metrics. The inset (adapted from Victor, 2005) shows an example of the series of operations taken to transform one pulse train into another by deleting (red), shifting (small blue arrows), or adding (green) spikes. The sum of the cost assigned to each operation determines the final cost estimate. The final step of the algorithm projects the pair-wise distance matrix into a low-dimensional similarity space **(C)**. Light blue outlines show how an individual pulse train is represented at each stage in the process. In the final output, the distance between the points represents the degree of similarity between the pulse trains. Note that this schematic is for illustrative purposes and does not contain real data.

Here, we utilize a novel algorithm for quantifying and comparing timing within sonar sound sequences. This algorithm, spike train similarity space (SSIMS), was originally formulated to compare the spiking patterns of neurons, in motor cortex recordings from rhesus macaques (*Macaca mulatta*) performing reaching and grasping tasks (Vargas-Irwin et al., 2015a,b). It combines point-process distance metrics and dimensionality reduction to compare spike train patterns to one another. Treated as time-series point processes, echolocation call sequences can be substituted for neural spike trains. Then the cost of transforming each biosonar sequence into each other sequence can be represented as the distance between the points on the SSIMS plot (**Figure 2**).

We applied the SSIMS algorithm to biosonar pulse trains emitted by bats flying through obstacle arrays. Mapping data into a SSIMS projection is done without any information regarding behavioral condition (unsupervised dimensionality reduction). Instead, the method relies on the intrinsic properties of the data and does not require an explicit model of the relationship between echolocation behavior and external variables such as the structure of the acoustic scene. One advantage of this method is that IPIs from sequences of echolocation pulses emitted by behaving bats can be compared without imposing absolute limits on what constitutes a sonar sound group (Kothari et al., 2014). This is essential because sonar sound group parameters appear to be dependent on behavioral context (Petrites et al., 2009; Falk et al., 2011; Kothari et al., 2014; Sändig et al., 2014; Knowles et al., 2015; Warnecke et al., 2015).

The first aim of this study was to test the effectiveness of the SSIMS algorithm in predicting bat biosonar behavior in a previously analyzed dataset (Wheeler et al., 2016). In this prior study, referred to here as Experiment 1, bats flew down an open corridor in the center of an obstacle array. This obstacle-free flight corridor varied in width from 100 to 70 cm, and 40 cm. The bats patterned their sounds differently depending on corridor width, so we used this dataset to test the ability of SSIMS to cluster call sequences made in each of the different corridor widths.

The second aim was to apply SSIMS to test the hypothesis that pulse trains recorded from bats flying in predictable (fixed) surroundings would be different from those recorded from bats challenged with unpredictable (variable) surroundings. This analysis was carried out using new, previously unpublished data. In Experiment 2, bats flew down the 40 cm wide flight path, which was manipulated into different shapes. When the flight path shape was fixed, bats could expect what the next flight path shape would be, but when the flight path shape was variable, the next flight path was unknown to the bats *a priori*. Previous studies have shown that over repeated exposure to the same flight environment bats develop stereotyped flight patterns (Barchi et al., 2013), but it is unknown how the structure of full-flight call patterns varies in response to unexpected changes in the environment.

## Materials and Methods

Echolocation calls were recorded from big brown bats (*E. fuscus*) in two experiments. Both experiments required bats to navigate through complex obstacle arrays that were expected to have some effect on the timing of their biosonar emissions. Experiment 1 was conducted between September 20 and November 27, 2013. Experiment 2 was conducted between April 4 and June 3, 2014. Timing of emitted biosonar sounds was measured and used to evaluate echolocation pulse trains with the SSIMS algorithm (**Figure 2**).

### Animal Subjects

**FIGURE 3:**
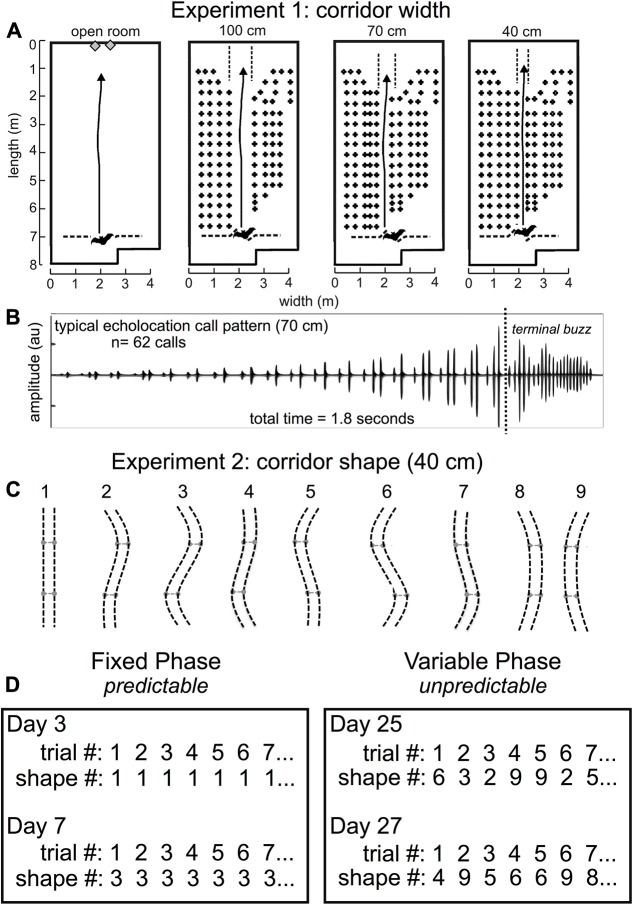
Experiment 1 and Experiment 2 methods. Flight room schematic for Experiment 1 **(A)**. The back of the room is at the top of each diagram, and the front of the room where the experimenter released the bat is shown at the bottom of each diagram. The active flight volume was filled with rows and columns of chains except for an obstacle-free flight corridor in the center of the chain array. In Experiment 1, the open room had no chains, or the corridor was straight and 100 cm, 70 cm, or 40 cm wide [**A**, adapted from Wheeler et al. (2016)]. Arrows point to the wall where the bat was trained to land. A typical echolocation call pattern in the 70 cm corridor **(B)** shows pulse patterns while the bat navigated the chain array, followed by the *terminal buzz* – a stereotyped, very fast call pattern that bats emit when landing or intercepting prey. Before pulse intervals were analyzed, the terminal buzz was excluded. Bats typically flew for 1.5–2.5 s to traverse the obstacle array before landing. In Experiment 2, **(C)**, the corridor was 40 cm wide and curved into 1 of 9 different configurations. Configuration 1 was identical to the 40 cm condition in Experiment 1 and was the starting point for all other configurations. The two movable crossbar joints (light gray) maintained consistent 40 cm spacing throughout the full corridor length and were moved 40 cm either to the right or to the left of center to create curved corridors of varying shapes, numbered 1–9. Experiment 2 consisted of two distinct phases: the fixed phase and the variable phase **(D)**. In the fixed phase, trials, or flights through the obstacle array, repeated the same corridor configuration up to 10 times in the same day for multiple days. In the variable phase, trials (flights) through the array varied pseudo-randomly each day. Actual trials are shown for experimental days (not consecutive days) 3, 7, 25, and 27. Adapted with permission from Accomando et al. (2017).

Four adult male big brown bats (*E. fuscus*), naïve to the laboratory flight room in Experiment 1, were trained to fly through an obstacle array (**Figure 3**). The bats were wild-caught (collecting permits #2012-34 and #2013-32, Rhode Island Department of Environmental Management). Bats were housed in a colony room (22–24°C, and 60–75% relative humidity) set to a 12-h/12-h reversed dark/light cycle in order to perform diurnal experiments on nocturnal bats. Bats ate enough larval *Tenebrio molitor* to maintain a mass between 15 and 18 g and had free access to vitamin-supplemented water (Poly-Vi-Sol, Enfamil) refreshed daily. The same four bats were flown in Experiments 1 and 2. The study was carried out in accordance with the recommendations of the Brown University Institutional Animal Care and Use Committee (IACUC). The protocol was approved by the Brown University IACUC.

### Flight Room Configuration

All experiments were conducted in a customized flight room (**Figure 3A**) that was insulated acoustically and electrically from outside noise. The flight room was covered in acoustic foam (Sonex “One” panels, West General LLC, San Jose, CA, United States) to absorb 20–25 dB of the sound energy from echolocation calls. An infrared camera (Photon 320, FLIR, Billerica, MA, United States) was positioned to view the bats in flight so that experimenters in the adjacent electronic control room could monitor in real time while controlling the sound-recording system. The flight room measured about 8 m long × 4 m wide × 3 m high, and the active flight area was 7 m long. Rows of multiple black plastic chains (Petrites et al., 2009; Barchi et al., 2013; Knowles et al., 2015) were hung from crossbars near the ceiling of the flight volume to act as quantifiable surrogates for vegetation-like acoustic reflectors (Petrites et al., 2009; Yovel et al., 2009). These chains spanned nearly the entire floor-to-ceiling distance, and acted as a dense obstacle matrix through which the bats could not fly except for an open flight corridor.

In Experiment 1, the chains were configured as shown in **Figure 3**. Rows of chains were spaced 20 cm apart, and chains within a row were spaced 30 cm apart. Rows of chains were added to either side of the 100 cm central corridor to create a 70- and a 40-cm-wide corridor. In this experiment, flight paths down the central corridors were straight. Detailed analysis of the results from this experiment (Wheeler et al., 2016) shows that the proximity of the nearest obstacles had a significant effect on call timing as measured by IPI and the ratio of pulse intervals following and preceding each call (post-IPI/pre-IPI ratio).

In Experiment 2, chains throughout most of the flight room were spaced identically to the 40 cm configuration in Experiment 1. The chains forming the central corridor were hung from flexible, 1/4″ diameter PVC pipes, which were secured to the ceiling to maintain a consistent corridor width of 40 cm. Two movable joints in these pipes were spaced 1.8 m apart and 90 cm from the midpoint of the chain corridor. The shape of this corridor was manipulated by positioning each joint in one of three positions (central, left, and right), allowing for a total of nine curved corridor configurations (**Figure 3**). Stoppers were placed 40 cm from the ends of each corridor edge so that the central joints would slide only 40 cm to either side, and the central position was marked such that the experimenter could move both joints to any of the three positions quickly and accurately. This allowed for a change in corridor configuration between each trial/flight while avoiding intrusion from chains located in neighboring rows.

### Experimental Procedure

Bats were released at the front of the room (**Figure 3A**) through an opening in a net and flew across the room to land on the back wall. As a control, bats also flew in the open room with no chains. Bats were rewarded with a mealworm for each successful flight. A successful flight was defined as when the bat navigated the obstacle matrix in its entirety without deviating from the corridor or crashing into or landing on one of the chains. Each bat was allowed a maximum of 10 flights per day. All flights were performed in the dark to exclude visual cues from the bats’ perception. A single long-wavelength (> 650 nm) red light allowed experimenters to find bats after each trial. In Experiment 1, bats flew down a 100-, 70-, and 40-cm-wide straight corridor in the flight room. **Figure 3B** shows a typical echolocation call pattern from the 70-cm-wide corridor. Notice that the time between echolocation calls (IPIs) decreases as the bat traverses the obstacle array and approaches the wall. Since the wall is the bat’s landing target, and the distance to the wall decreases as the bat approaches it, pulse intervals long enough to allow echoes to return from the far wall become shorter. The amplitude of the calls shown in **Figure 3B** increases because the bat is approaching the microphone used to record echolocation sounds. Echolocation calls in this example are primarily grouped into doublets, or sonar sound groups having two calls within a group.

Experiment 2 used the same 40-cm-wide corridor from Experiment 1, but the path shape varied (**Figure 3C**). Experiment 2 was conducted in two broad phases: the fixed phase and the variable phase. **Figure 3D** shows example trials from both phases of Experiment 2. This experiment was designed this way in order to determine whether expectation could influence the timing of biosonar calls emitted by the bats. In the fixed phase, bats could expect the same corridor configuration from trial to trial; but in the variable phase, bats could not predict the next corridor configuration after each trial. The hypothesis was that SSIMS could differentiate predictable, expected (fixed) flights from unpredictable, unexpected (variable) flights based on biosonar sound timing patterns.

In the fixed phase, the configuration of the corridor did not change from trial to trial. Bats flew down the same configuration for up to 10 successful trials per day. First, the corridor was fixed at 40 cm wide, and bats flew down a straight corridor (shape 1) for 5 days. Then, bats were trained to fly down a partially curved corridor (shape 2) for 1 day before flying down a maximally curved corridor (shape 3) for 3 days. Next, bats were trained to fly in shape 5 for 1 day, then a full S-curve (shape 6) for several days. In each of these conditions, the corridor did not change from trial to trial within a day. A total of 470 trials or flights were successfully completed in the fixed phase, while 2.9% (*n* = 14) of all attempted flights (*N* = 484) were unsuccessful.

In the variable phase, the corridor configurations changed from trial to trial. In this phase, the bat was transferred from the colony room into the flight room in an acoustically insulated box so that the bat could not scan the room upon entering and before being released for flight. In preparation for releasing the bat into the chain array, the experimenter held the bat in one hand, and with the other hand, held a large piece of acoustic foam in front of the bat until the moment of release. This precaution was taken to prevent the bat from gathering pre-flight information about the configuration of the chains. The variable phase was conducted in two parts. In the first part, the corridor conditions were pseudo-randomly varied between configurations 1, 3, and 6. In the second part, the corridor configuration varied pseudo-randomly between all nine possible shapes (**Figure 3C**). The randomization did not allow more than one repeat of the same configuration from flight to flight. Both parts of the variable phase were combined for analysis. Fewer flights were conducted in configurations 2, 4, 5, 7, 8, and 9 than the other configurations, so no comparison was made for these configurations in the fixed versus variable phases. Shapes 4, 5, 7, 8, and 9 were only presented in the variable phase. All corridor configurations were new to the bats in Experiment 2 with the exception of shape 1. The flight path shapes were pseudo-randomly presented to decrease the probability that bats could predict which configuration they would be required to navigate. A total of 379 successful trials were recorded, and 2.8% (*n* = 11) of all attempted flights (*N* = 390) were unsuccessful.

### Sound Recording

Two electret ultrasonic microphones (MEMS SPM0404UD5, Knowles Electronics, Itasca, IL, United States) were mounted on custom preamplifier and high-pass filter boards that were located on the far wall directly in front of the corridor exit. To minimize backscatter from the wall, behind each microphone-preamplifier unit was a square, 20-cm foam baffle. These microphones detected sounds emitted by bats during flight (gray diamonds, **Figure 2**). Outputs of the 2 channels were digitized (one PCIe-424 with two accompanying HD192, all from MOTU, Cambridge, MA, United States) at a sampling rate of 192 kHz and stored as 16-bit wav files. All electronic wiring from microphones and the infrared camera were fed through a small opening to an adjacent control room. Experimenters in the control room began the sound recording when instructed by the experimenters in the flight room, and stopped recording when the bat landed on the far wall as visualized in real time by the infrared camera. Using this procedure resulted in sound recordings that included some echolocation calls from before the bat entered the chain array, and after the bat exited the chain array.

### Pulse Interval Analysis

Sounds recorded by the microphone that had the best consistency of echolocation signal amplitude throughout each successful flight were analyzed for IPIs. The single-channel recordings used in these experiments were always from one of two microphones located in the center of the far wall. The higher amplitude recording was chosen from the two possible microphones. All wav files were truncated to include only in-flight sounds (**Figure 3B**), which removed the terminal buzz and any pre-flight calls. A custom program (MATLAB, MathWorks, Cambridge, MA, United States) identified and low-pass filtered frequencies below 35 kHz to exclude communication sounds, and high-pass filtered frequencies above 50 kHz to include only ultrasonic echolocation calls. The envelope of each sound was computed using the Hilbert transform. The program extracted IPIs from the time between peak amplitudes for successive sounds. To exclude any echoes or sounds emitted outside of the flight, only IPIs between 12 and 100 milliseconds were included in subsequent analysis.

### SSIMS Algorithm Implementation

Details of the original SSIMS algorithm can be found in a previous publication (Vargas-Irwin et al., 2015a), but are summarized in **Figure 2**. The SSIMS algorithm embeds point-process data into a high-dimensional pair-wise similarity space, and then projects the data into a more compact (low-dimensional) representation, which facilitates statistical analysis as well as data visualization while still capturing the relationship between individual data points. Originally designed for neural data analysis, SSIMS uses the spike train metric proposed by Victor and Purpura (1996) to quantify similarity (Victor, 2005). The method evaluates differences in spike trains based on how spikes in one train have to be shifted, deleted, or inserted to make it precisely match another train (**Figures 2A,B**). This cost function is similar to the “edit distance” or “Levenshtein distance” used to compare letter strings or genetic sequences. The algorithm can be directly applied to bat biosonar pulses by treating each pulse as an action potential. This is equivalent to treating each bat as a single neuron, and each flight down the corridor as a spike train recorded from a neuron in response to a stimulus.

The use of spike train metrics makes it possible to analyze long time periods (on the order of seconds) while preserving the fine temporal structure of call patterns. Note that only the timing of each pulse is considered and not the spectral characteristics. The cost of shifting a pulse by 20 ms was equivalent to inserting and deleting a pulse (1/*q* = 20 ms). This value for 1/*q* was arrived empirically – SSIMS achieved optimal classification performance when 1/*q* = 20 ms. Different values of *q* resulted in qualitatively similar results, but with slightly lower classification values (data not shown). The high-dimensional space defined by pair-wise distances is projected into a low-dimensional representation *t*-distributed stochastic neighbor embedding (*t*-SNE) (**Figures 2B,C**) (van der Maaten and Hinton, 2008). This method is well suited to this type of analysis because it is based on pair-wise similarity estimates and explicitly seeks to preserve the structure within local neighborhoods – in this case, clusters of flights with similar call patterns. A single point in the resulting SSIMS projection represents the timing of echolocation pulses recorded during a specific flight (**Figure 2C**). The distance between points represents the degree of similarity between the call patterns they represent. Two identical call patterns correspond to the same point in this space; the more different they are, the farther apart they lie in the SSIMS projection. Projecting the data into a low-dimensional space makes it easy to visualize the similarity between different experimental conditions in a way that also displays information about each individual flight. Dimensionality reduction also increases the density of the data (relative to the dimensionality of the space), making it easier to characterize data distributions and perform pattern recognition (i.e., classification).

## Results

The SSIMS approach (**Figure 2**) categorized streams of bat echolocation pulses (**Figure 1**) from individual flights in two experiments (**Figure 3**). Both experiments were conducted in a flight room with plastic chains acting as an obstacle array. Experiment 1 used three different corridor widths to test the effect of obstacle proximity on biosonar sound patterning. Experiment 2 tested the effect of the bats’ expectation on call patterns using predictable (fixed) and unpredictable (variable) corridor shapes. See Supplementary Infrared Video for an example flight down the straight 40 cm corridor.

### Experiment 1

**FIGURE 4:**
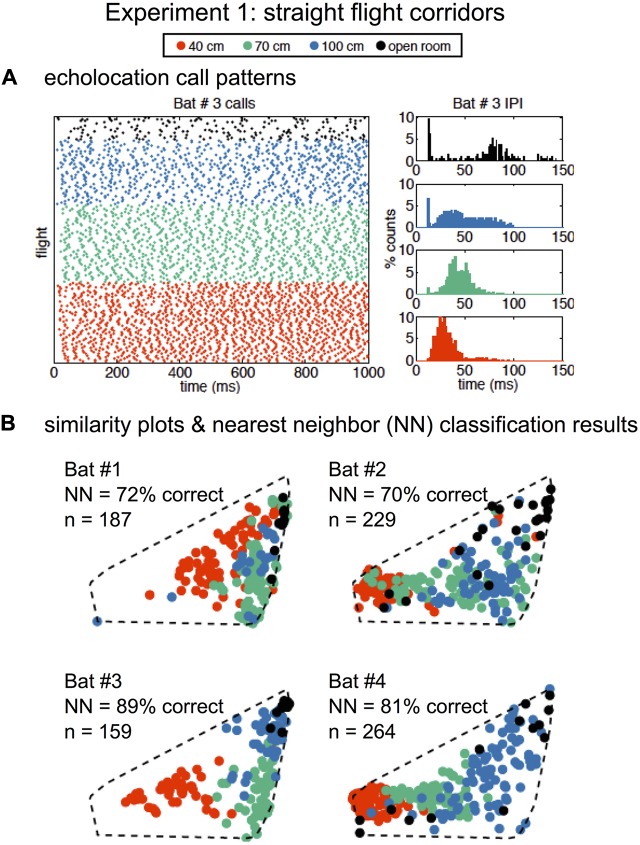
Similarity analysis reveals environment-dependent variation in call patterns. Raster of all recorded echolocation calls (points) over all of Bat #3’s flights (rows) (**A**, left panel). Inter-pulse interval (IPI) histograms (**A**, right panels) for Bat #3 in four conditions: open room (black), 100 cm corridor (blue), 70 cm corridor (green), and 40 cm corridor (orange). IPIs were shorter and more calls were emitted with increasing clutter. Similarity plots separated by bat are shown in **(B)**. Each point represents a flight (over the same time interval presented in **A**), and the distance in the 2D space represents the estimated similarity between the call patterns. The similarity space was generated using calls recorded from all bats from 839 flights total. Nearest-neighbor (NN) classification results (10D similarity estimates) were calculated individually for each bat using leave-one-out cross validation. In all cases, classification exceeded expected chance levels (the upper limit of the 99% confidence interval obtained empirically from 1000 random label permutations was 46%). Dotted lines show that all bats are plotted in the same similarity space for ease of comparison. Adapted with permission from Accomando et al. (2017).

In Experiment 1, SSIMS analyzed sequences of pulses from individual flights, and the resulting SSIMS plots (**Figures 2C**, **4B**) allowed clusters of points (call sequences or flights) to be classified as belonging to the open room, and the 100-, 70-, and 40-cm-wide corridors in the obstacle array. Calls from all bats were projected onto a single common similarity space (dotted line in **Figure 4B**) such that the representation of each call was calculated using similarity estimates across the entire set of recordings. Raster plots in **Figure 4A** display data from one example bat to illustrate that bats emitted more calls with shorter IPIs (histograms on the right) as obstacle array clutter increased and flight corridor width decreased. Histograms in **Figure 4A** show that the distribution of IPIs shifted toward shorter time intervals with decreasing corridor width, indicating that more calls were made in groups.

We quantified the differences between call patterns using a nearest-neighbor (NN) classifier implemented using leave-one-out cross validation. The NN classifier was chosen because it is designed to capture the local structure of complex data patterns, making it well suited to interpret data processed using SSIMS (which is designed to preserve exactly this kind of relationship). Classification was performed using 10D SSIMS projections. Asymptotic performance was achieved with between 2 and 6 dimensions for different bats (see Supplementary Figure 1). NN classification correctly assigned flights (*N* = 839) with 70–89% accuracy depending on the individual bat (**Figure 4B**).

In Experiment 1, all bats decreased the mean IPI as more obstacles were added to the flight room (see **Table 1**). Comparing the open room flights to the 40-cm-wide corridor flights, bats decreased the length of the mean IPI by 29–62% (a decrease of 13–44 ms), depending on the bat.

**Table 1:** Mean inter-pulse interval (IPI) in milliseconds for Experiment 1 flights.

	Open room	100 cm	70 cm	40 cm
Bat 1 Mean (*SD*)	52 (41)	40 (18)	39 (17)	36 (17)
Bat 2 Mean (*SD*)	70 (44)	32 (15)	29 (13)	26 (13)
Bat 3 Mean (*SD*)	44 (52)	39 (21)	37 (16)	31 (12)
Bat 4 Mean (*SD*)	41 (70)	31 (16)	34 (12)	24 (11)


#### Comparison of Experiment 1 SSIMS Analysis with Previous Analysis

Previous analysis of the Experiment 1 dataset (Wheeler et al., 2016) looked at IPI distributions, sonar sound grouping as defined by Kothari et al. (2014), and the bimodal distribution of IPI ratio, defined as the ratio of the interval of time following a call to the interval of time before that same call (post-IPI/pre-IPI) (Wheeler et al., 2016).

##### Experiment 1 previous IPI analysis compared to SSIMS

The IPIs between calls emitted by all four bats in the three corridor widths were compared using a generalized linear mixture model (GLMM). This analysis showed significant differences between the 40 and 70 cm corridor (*p* < 0.0001) and 40 and 100 cm corridor (*p* < 0.0001). However, no significant differences were found between mean IPI in the 70 and 100 cm conditions. The clustering of green points (70 cm) and blue points (100 cm) in the SSIMS projections in **Figure 4B** shows that using the entire timing pattern enhances the visibility of differences between these two conditions that the mean IPI comparison does not capture. The GLMM revealed that the bats decreased their IPIs as they approached the wall at different rates depending on corridor width (*p* < 0.0001 for all comparisons), and part of this dependence was the result of significantly different IPIs from the beginning of the flight (*p* < 0.0001, 40 cm compared to 100 and 70 cm) (Wheeler et al., 2016). These two features of temporal differences between corridor widths were likely captured by the SSIMS method.

##### Experiment 1 previous sonar sound group analysis

In the prior analysis of Experiment 1 data, sonar sound groups [defined as having a stable within-group IPI, 5% tolerance, and a between-group IPI at least 1.2 times greater than the within group IPI (Kothari et al., 2014)] were compared across corridor widths using a Chi-square analysis. Sonar sound group analysis found singles, doublets (groups of 2 sounds), triplets (groups of 3 sounds), and quadruplets (groups of 4 sounds). No statistically significant difference was found in the proportion of sounds categorized into these groups depending on corridor width (Wheeler et al., 2016). However, across bats, trends revealed an increase in the percentage of singles, triplets, and quadruplets as the corridor width narrowed. The percentage of doubles showed the opposite trend – there was a decrease in the proportion of doubles as the corridor width became narrower.

The sonar sound group analysis showed that in the 40 cm corridor, bats used singles 44.6% of the time, doubles 37% of the time, triplets 16% of the time, and quadruplets 2.4% of the time. IPIs within sonar sound groups and between sonar sound groups became shorter with decreasing corridor width. The increased percentage of calls categorized as singles in the 40 cm corridor was not expected because it is thought that bats increase sonar sound grouping behavior in clutter, which logically would result in a decrease in “single” calls – those separated by relatively long time intervals before and after. However, this extremely dense obstacle array with very close proximity obstacles may have resulted in sound patterns that impeded the separation of sonar sound groups using the Kothari et al.’s (2014) method, thus resulting in the observed increase in singles.

Although the SSIMS method does not directly address the question of how sonar sound group parameters vary in specific circumstances, SSIMS uses the information contained in the timing patterns therein to estimate call pattern similarity over the entire flight time.

##### Experiment 1 previous IPI ratio analysis

To circumvent the problem of imposing absolute limits on what defines a sonar sound group, our previous study used the ratio of post-call IPI to pre-call IPI, or IPI ratio. The IPI ratio is a simple metric that can quickly estimate whether a call is likely a part of a sonar sound group. Previously, we used a finite mixture model (FMM) to determine how IPI ratio depended on corridor width. We found that the IPI ratio was a bimodal distribution where one of the distributions depended on corridor width, and the other did not (Wheeler et al., 2016). This suggests that some calls functioned to avoid obstacles, whereas others had some other function such as providing contextual information about the rest of the room, for example, the distance from the back wall.

#### Summary of Experiment 1 Results

In summary, the GLMM of IPIs in Experiment 1 did not show differences in mean IPI between the 70 and 100 cm corridor, though SSIMS clearly plotted these flights into different clusters (**Figure 4B**), and the NN method classified between 70 and 89% of flights into the corridor width accurately depending on bat. The sonar sound group analysis did not show significant differences between the proportions of singles, doubles, triples, and quadruples as a function of corridor width. The sound group analysis allowed us to determine that the IPIs shortened both within and between sound groups as the corridor width became narrower. SSIMS analysis did not provide specific information about sonar sound groups, but they certainly impacted the overall sound patterns that SSIMS plotted and the NN analysis categorized. Finally, the IPI ratio analysis suggested that the temporal pattern of biosonar sounds emitted by bats is determined by the nearest obstacle proximity and other unknown factors.

### SSIMS Analysis of within and between Bat Variability in Experiments 1 and 2

**FIGURE 5:**
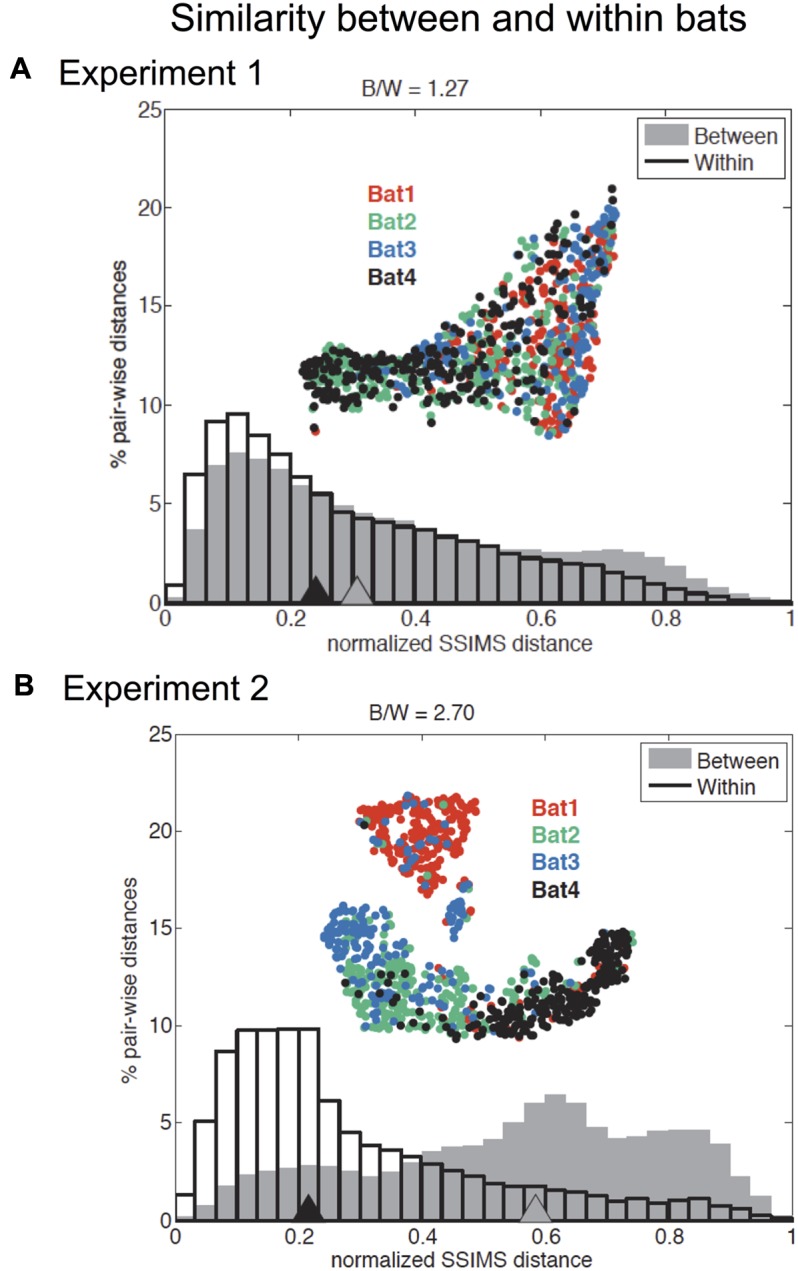
Effect of individual bat on temporal pattern similarity for Experiments 1 and 2. Comparison of normalized flight-by-flight pair-wise SSIMS distance within (black outline) and between (gray) bats for Experiment 1 **(A)** and Experiment 2 **(B)**. Triangles indicate medians. Insets display the SSIMS projections, colored by bat (red = Bat 1, green = Bat 2, blue = Bat 3, and black = Bat 4). There was a significant difference in medians for between and within bats comparisons in both experiments (KW *p* < 0.0001). The ratio of between bats to within bats median SSIMS distance (B/W) is reported at the top of each plot. The B/W ratio is a measure of the effect size. The effect was more pronounced for Experiment 2: The ratio of between to within bat call pattern distances was 2.70 for Experiment 2 compared to 1.27 for Experiment 1. The normalized between-bat distances were significantly higher (KW *p* < 0.0001) for Experiment 2 (comparing the two gray distributions in **A** and **B**). Normalized within bat distances for Experiment 2 are significantly smaller than in Experiment 1 (KW *p* < 0.0001). Adapted with permission from Accomando et al. (2017).

The recorded call sequences revealed that two pairs of bats – Bats 1 and 3, and Bats 2 and 4 – were more alike than to bats in the other pair (**Figure 5**). The differences in the call patterns employed by different bats were evaluated by comparing distributions of pair-wise SSIMS distances over all flights between and within bats. Statistical significance was assessed using a Kruskal–Wallis (KW) test, while the effect size was summarized using a ratio of SSIMS distance medians (distance between biosonar sound sequences – points – in similarity space) (B/W ratio). For example, a B/W ratio equal to 2 indicates that the normalized median distance between call patterns across different bats was twice of that observed for call patterns for the same bat. **Figure 5** shows that across all bats, the B/W ratio was 1.27 for Experiment 1 and 2.70 for Experiment 2. Although both values are statistically significant (KW *p* < 0.0001), the more than twofold difference in B/W ratio suggests an increase in bat-specific call pattern differences with increasing task difficulty.

To test whether within bat variability and between bat variability were significantly different between the two experiments, we performed a KW test, and found that normalized within bat distances for Experiment 2 were significantly smaller than in Experiment 1 (KW *p* < 0.0001). This result suggested that bats used more stereotyped call patterns in Experiment 2, which is likely because of the consistent 40 cm corridor width in this experiment. Additionally, the normalized between bat differences were significantly greater for Experiment 2 (KW *p* < 0.0001). This provided statistical support of our assertion that there were increased bat-specific differences in Experiment 2.

Pairs of bats were also analyzed for individual similarities, and only one pair was found not to be significantly different (KW *p* > 0.05): bat 2 and bat 4 in Experiment 1, suggesting that this pair of bats emitted very similar sound patterns for this experiment. All other bat pairs showed statistically significant differences for both Experiment 1 and Experiment 2 (KW *p* < 0.0001) (see Supplementary Figure 2).

### Comparison of Experiment 1 40 cm Flights with Experiment 2 Flights

**Tables 2**–**4** address the differences in mean IPI, mean number of calls per second, and the average flight velocities in the 40-cm-wide straight corridor during Experiment 1 versus the fixed and variable phases of Experiment 2. The columns in **Tables 2**–**4** are organized from left to right in the chronological order in which the experiments were conducted. The trends show that bats emitted calls having progressively longer mean IPIs (**Table 2**), fewer calls per second (**Table 3**), and slower flight speeds (**Table 4**) in the 40-cm-wide corridor as the experiments progressed. Between the straight 40 cm corridor flights in Experiment 1 and the variable phase of Experiment 2, bats decreased their average flight velocities by 11–35% depending on the individual bat (see **Table 4**). This could explain how bats navigated through more complex, unpredictable path shapes using fewer calls per second and longer mean IPIs. However, gross differences in mean IPI, calls/sec, and flight velocity between the fixed and variable phases of Experiment 2 were not readily apparent (see **Tables 2**–**4**). It could therefore not be determined from these parameters alone whether expectation had any effect on bat biosonar call patterning.

**Table 2:** Mean inter-pulse interval (IPI) in milliseconds for 40 cm corridor flights.

	Experiment 1	Experiment 2	
		
	40 cm wide straight corridor	Fixed phase	Variable phase
Bat 1 Mean (*SD*)	36 (17)	43 (22)	41 (21)
Bat 2 Mean (*SD*)	26 (13)	31 (12)	33 (12)
Bat 3 Mean (*SD*)	31 (12)	35 (14)	37 (14)
Bat 4 Mean (*SD*)	24 (11)	27 (10)	27 (10)


**Table 3:** Mean number of calls per second in 40 cm corridor flights.

	Experiment 1	Experiment 2	
		
	40 cm wide straight corridor	Fixed phase	Variable phase
Bat 1 Mean (*SD*)	26 (4)	24 (2)	25 (2)
Bat 2 Mean (*SD*)	40 (3)	33 (3)	30 (2)
Bat 3 Mean (*SD*)	32 (7)	30 (2)	27 (2)
Bat 4 Mean (*SD*)	42 (3)	39 (3)	38 (2)


**Table 4:** Mean average flight velocity^∗^ in meters per second during 40 cm corridor flights.

	Experiment 1	Experiment 2	
		
	40 cm wide straight corridor	Fixed phase	Variable phase
Bat 1 Mean (*SD*)	3.8 (0.9)	3.1 (0.5)	3.0 (0.4)
Bat 2 Mean (*SD*)	3.7 (0.8)	2.6 (0.4)	2.4 (0.2)
Bat 3 Mean (*SD*)	3.4 (0.7)	3.3 (0.6)	3.0 (0.2)
Bat 4 Mean (*SD*)	3.7 (0.5)	3.2 (0.4)	3.2 (0.3)


**^∗^Flight velocity was averaged across each entire flight, and then, the mean of all flights in each condition is reported*.*

### SSIMS Analysis of the Effect of Expectation on Biosonar Call Patterns

To evaluate whether expectation can impact the timing of active sonar probing by bats, SSIMS was used to assess call patterning in two phases of Experiment 2. In order to compensate for the greater variability in call patterns across bats for Experiment 2, (see Supplementary Figure 2) individual similarity spaces were generated for each subject such that a call pattern was represented in terms of the relationship to calls emitted by the same bat. In this experiment, the same four bats from Experiment 1 flew down 40-cm-wide corridors of varying shapes (**Figure 3**). In the fixed phase of Experiment 2, the bats flew down corridors having the same configuration from trial to trial. In the variable phase of Experiment 2, the corridor configuration was pseudo-randomly changed from trial to trial (see **Figure 3D** and see section “Materials and Methods”).

**FIGURE 6:**
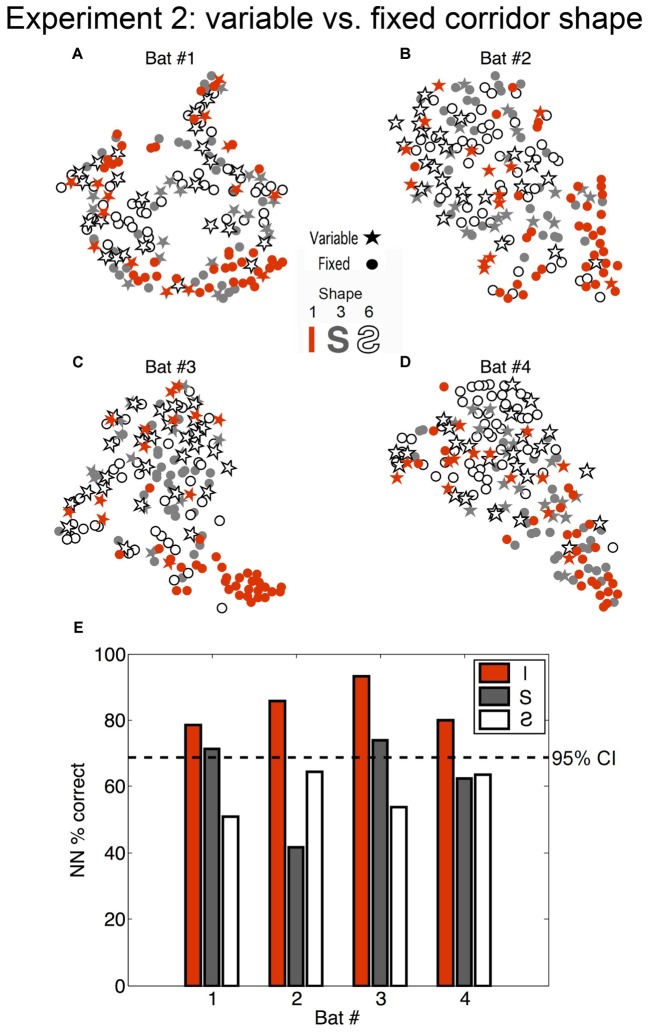
Effect of bat expectation on echolocation behavior. **(A–D)** Each star or dot represents a flight where the corridor configuration was straight (orange) (Configuration 1 in **Figure 2**), an S-curve (gray) (Configuration 6), or a reverse-S-curve (white with black outline) (Configuration 3). Dots represent flights from the fixed phase of Experiment 2, where the bats (**A** = Bat #1, **B** = Bat #2, **C** = Bat #3, **D** = Bat #4) each flew down the same corridor for 10 trials per day for several days. Stars represent flights that occurred in the variable phase of Experiment 2, where the corridor configuration was variable (i.e., unexpected) and changed pseudo-randomly from trial to trial. Flights in the unexpected straight-corridor (orange stars) had echolocation pulse trains that were classified as more similar to flights in the curved corridors than expected flights in the straight corridor (orange dots). **(E)** Histogram showing nearest-neighbor (NN) percent correct classification for each configuration and each bat (performed on 10D similarity estimates using leave-one-out cross validation). Dotted line represents the upper 95% confidence limit of the chance distribution (obtained empirically from 10,000 random label permutations). Histogram bars that cross the dotted line represent successful categorization of whether the flight occurred in the fixed or variable phase of Experiment 2. Adapted with permission from Accomando et al. (2017).

To determine whether the bats’ expectation of the corridor shape had any effect on echolocation behavior, the flights down the straight and S-curved corridors (shapes 1, 3, and 6, see **Figure 3C**) in the fixed phase and variable phase were compared. We evaluated differences between the fixed and variable conditions using a NN classifier. Fixed versus variable classification results were inconsistent for the S-curve shapes (exceeding chance in only 2 out of 8 comparisons), but were consistently above expected chance levels in the straight corridor configuration for all bats. **Figure 6** shows that (1) during fixed straight (shape 1) flights bats produced stereotyped call patterns different from the curved corridor flights; and (2) that flights down the straight corridor during the variable phase were more like curved corridor flights than fixed phase straight corridor flights. In other words, unexpected straight flights were more like curved flights than expected straight flights.

The absence of clustering for all variable flights from all fixed flights (stars and dots, respectively, in **Figure 6**) suggests that expectation, or the ability of the bat to predict the flight path shape, did not determine overall temporal patterns emitted in the chain array. Rather, SSIMS plotted clusters of straight flights in the fixed phase apart from all other flights. Our results showed that the bats altered their call patterns in a similar way to address (1) more complex flight paths and (2) unpredictable flight paths. This finding suggests that bats adopted a more conservative (careful) strategy whenever a more challenging or unknown situation was presented.

### IPI Ratio Distribution Analysis

**FIGURE 7:**
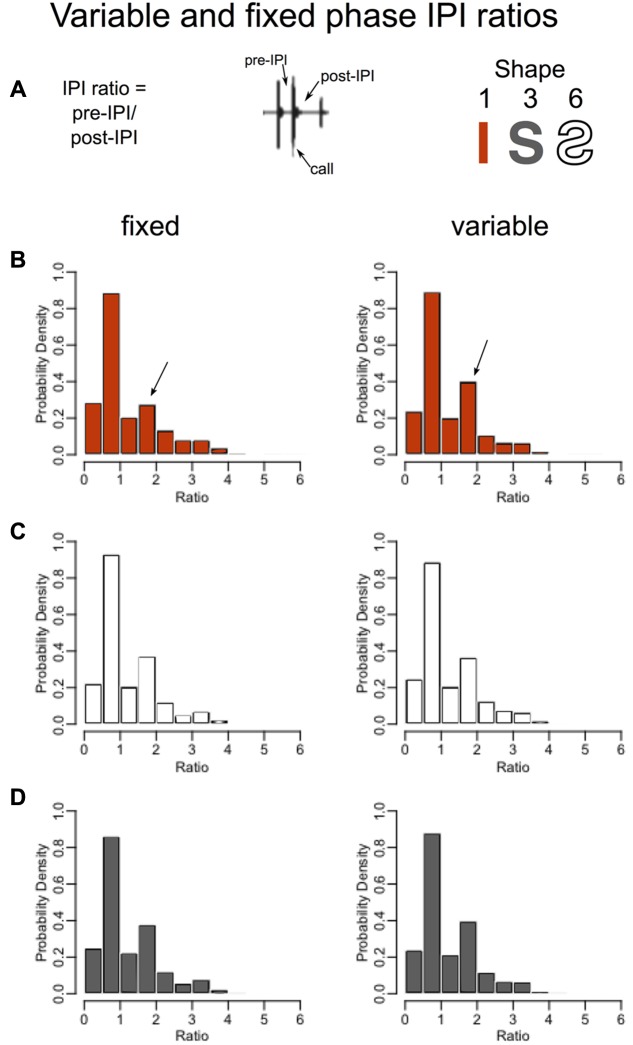
Higher probability of sonar sound grouping in unexpected straight flights. Without imposing limits on what constitutes a sonar sound group, one can look at the probable grouping of sounds by calculating the IPI ratio, or the pre-call time interval divided by the post-call time interval (Ratio = pre-IPI/post-IPI) **(A)** (Wheeler et al., 2016). Histograms in **B–D** show the proportions of IPI ratios for the same flights as in **Figure 6**. The fixed phase or expected flights are plotted in the left panels while the variable phase unexpected flights are plotted on the right. Straight corridor (shape 1) flights are in red **(B)**, the reverse-S-curve flights (shape 6) are plotted in white, and the S-curve flights (shape 3) are plotted in gray **(D)**. In the straight corridor configuration **(B)**, bats emitted a greater proportion of calls having a post-IPI/pre-IPI ratio of 1.5–2 (arrows). The interval of time following these calls is roughly 1.5 times longer than the interval preceding these calls, which suggests that these calls represent the last call in a sonar sound group. The increase in proportion of these calls from the fixed to the variable phase indicates that bats use more sonar sound groups in unpredictable surroundings.

To identify which features the SSIMS analysis may have detected to discriminate fixed versus variable straight flights but not fixed versus variable curved flights, the post-IPI to pre-IPI ratios and IPI distributions were plotted (**Figure 7**). In the straight corridor configuration (red), bats emitted a greater proportion of calls having a post-IPI/pre-IPI ratio between 1.5 and 2. Arrows in **Figure 7B** show where this is visible on the histograms. This ratio value indicates that the interval of time following these calls was roughly 1.5–2 times longer than the interval preceding the calls, which means that they likely represent the last call in a sonar sound group (Kothari et al., 2014; Wheeler et al., 2016). **Figure 7** indicates that the bats in this experiment used more sonar sound groups in unpredictable learned surroundings (variable, straight path) than predictable learned surroundings (fixed, straight path). The inability of SSIMS to reliably categorize fixed versus variable curved flights (**Figure 6**), and the absence of visible differences in the IPI ratios for these flights (**Figures 7C,D**) provides evidence that memory plays a more important role than expectation in biosonar sound patterns emitted by big brown bats.

The SSIMS method ignores sonar sound groups entirely, and provides a clear visualization of the magnitude of the difference between call patterning in the fixed and variable flights as compared to the IPI ratio histograms (compare **Figures 6**, **7**).

## Discussion

The results of the SSIMS analysis (1) confirm that the bat’s echolocation call sequences are influenced by the proximity of obstacles in the surrounding environment (Petrites et al., 2009; Wheeler et al., 2016), (2) demonstrate that individual bats’ strategies for navigating become more distinct as the difficulty of the task increases, and (3) demonstrate that previous training in the flight path influences temporal patterning of bat echolocation calls.

**Figure 4** shows that the SSIMS algorithm was able to use intrinsic information of the echolocation call patterns from individual flights in the first experiment (Experiment 1, **Figure 3**) to predict in which of the three conditions the bats were flying. These results are consistent with previous findings that bats change echolocation pulse timing in response to obstacles or environmental clutter (Petrites et al., 2009; Moss and Surlykke, 2010; Falk et al., 2014; Kothari et al., 2014; Wheeler et al., 2016). Furthermore, this work provides confirmation that the overall temporal patterning of flights in close-proximity clutter is dependent upon the distance to the obstacles (Petrites et al., 2009; Kothari et al., 2014; Wheeler et al., 2016). The SSIMS method and subsequent NN classification showed differences between the 70 and 100 cm corridor flights, while a GLMM of the mean IPIs was unable to do so (Wheeler et al., 2016).

Significant differences between individual bats were found in both experiments. **Figure 5** shows the difference between mean normalized SSIMS distance between bats was significantly greater than within bats for both tasks. However, this distance more than doubled for Experiment 2. Since this experiment required more complex aerial maneuvering through curved flight corridors of only a 40 cm width, we conclude that the increase in between-bat SSIMS distance reflects increased bat-specific differences with a rise in task complexity or difficulty.

**Figure 6** shows that previous experience in the flight path influenced biosonar timing in Experiment 2. In the fixed phase of Experiment 2, call patterns emitted by bats flying in the curved corridors clustered apart from those emitted in the straight corridor. In the variable phase, unexpected straight corridor flights clustered more closely with expected curved corridor flights than expected straight corridor flights. This suggests that, although flights down the straight corridor were identical, navigating them was more difficult for the bats when they were not predictable or expected. Call patterning was influenced both by the shape of the corridor (curved or straight) and by the opportunity the bats had to predict their environment before flights. This is consistent with a previous finding that big brown bats change their echolocation emission patterns in response to a randomly positioned barrier earlier when the presence of the barrier was unexpected than when it was expected (Knowles et al., 2015). Past research has also shown that bats tracking a moving target while sitting stationary on a platform exhibited distinctive call pattern changes when tracking a target that moved unpredictably compared to predictably (Kothari et al., 2014). The absence of reliable SSIMS clustering for expected versus unexpected flights in the curved corridor shapes, shown in **Figure 6**, combined with the fact that bats were not trained as extensively in these path shapes, suggests that memory or experience was more important than expectation as a cognitive factor. If expectation alone caused the differences seen in the variable versus fixed straight corridor condition, then this effect would also be present for curved corridor flights; but it was not consistently observable for those path shapes (see **Figure 6**).

Together, these studies show that big brown bats anticipate the challenges of a familiar environment and adapt their echolocation pulse patterning to accommodate unexpected changes. The reliability of SSIMS in detecting differences in the echolocation pulse patterns by corridor width (**Figure 4**), and the comparatively less reliable classification of pulse patterns by expectation (**Figure 6**) showed that the proximity of the nearest obstacles – or physical nature of the environment – was more predictive of pulse patterning than cognitive state.

### Advantages and Limitations of the SSIMS Method

The advantage of using SSIMS to analyze temporal patterning of bat echolocation sounds across a flight is that the effect of an environment on the timing of vocalizations can be visualized easily. The major limitations of this method for such analyses are as follows: (1) other aspects of the echolocation signal such as duration, frequency spectra, and beamform are not incorporated into the similarity estimates; and (2) finer temporal details about sonar sound group production, while included in the analysis, are not explicitly reported on the SSIMS space projection.

SSIMS representations are useful in determining the degree to which echolocation call sequences are similar or different. However, SSIMS alone cannot pinpoint individual timing features such as sonar sound groups, flight velocity, IPIs, and IPI ratios. SSIMS analysis also ignores all other adaptable features of biosonar pulses, including frequency and amplitude.

SSIMS can provide a valuable initial screening such that further examination of individual features that may contribute to SSIMS distances is more directed. The outline of the previous analysis of Experiment 1 (see section “Results”) exemplifies the arduous process of finding meaningful pulse timing differences using other methods. Using SSIMS, biosonar sound sequences from an entire flight event can be represented in a single point, and easily compared to other flights on the same plot. This ease of visualization is advantageous because it reveals which comparisons of individual timing features might be most interesting.

### Comparison with Other Analysis Methods

The advantage of SSIMS over other methods such as linear mixed effects models (Falk et al., 2015; Wheeler et al., 2016), Markov Chain analysis (Petrites et al., 2009; Kloepper et al., 2014), and others, that it requires no boundaries or binning and very few assumptions. It preserves fine temporal structures (millisecond scale) even over relatively long time scales (second or minute scale).

Perhaps the greatest advantage of SSIMS is that the resulting scatter plots in SSIMS space are easy to interpret visually. For example, the SSIMS plot in **Figure 6** clearly shows that expected straight corridor flights are different from unexpected flights in the same path. The **Figure 7B** histogram shows that the post- to pre-IPI ratio between 1.5 and 2 increases when the straight corridor flights are unexpected. **Figure 7B** does not illustrate differences in IPI ratio distributions for the curved flight paths. While **Figure 7** shows an example of what types of differences SSIMS is detecting, the degree to which pulse patterning changes is more easily visualized in the similarity plot.

Past research has diligently characterized individual echolocation sound parameters (duration, mean IPI, repetition rate, starting and ending FM sweep frequencies, etc.,) that adapt to obstacle avoidance or target-detection conditions. Take, for example, sonar sound groups. Sonar sound groups, or strobe groups, are loosely described as a group of sounds having short time intervals between calls within the group, and longer time intervals delineating the group (Moss et al., 2011; Kothari et al., 2014). More specifically, these groups have been mathematically defined as having at least a 20% longer interval between groups (“island criterion”), and no more than 5% within-group variability (“stability criterion”) (Kothari et al., 2014). While useful for some classification purposes, these limitations are arbitrary, and have made it difficult to compare pulse interval patterning across experiments. This is partly because IPI values alternate between long and short to define sound groups, and these absolute time measurements appear to be scaled proportionally to the environment (Moss et al., 2006, 2011; Petrites et al., 2009; Aytekin et al., 2010; Kothari et al., 2014; Wheeler et al., 2016). This proportionality extends over a wide enough range in big brown bats that the interval categories of short versus long overlap. Thus, using set time interval sizes or too narrow a range of size ratios to define sound groups does not fully capture the bat’s behavior. In contrast, SSIMS can determine whether a particular environment or condition resulted in an observed change in echolocation call timing, without *a priori* restrictions.

## Conclusions and Future Directions

The research presented here (1) demonstrates the utility of SSIMS for analyzing echolocation sound sequences, and (2) implements SSIMS to evaluate changes in biosonar patterns due to changing environmental complexity, and as a result of the bats’ expectation of the flight path through the obstacle array. The SSIMS method streamlines efforts to quantify biosonar behavior because it provides simple visualization and flexible quantification of temporal pattern differences. The SSIMS method was developed to analyze neural spike timing patterns, and it is a powerful, unbiased approach for comparing neural data (Vargas-Irwin et al., 2015a). In fact, the SSIMS method is a general technique widely applicable to all kinds of time-series data.

No direct quantitative comparisons between SSIMS and other classification methods were made in this study. Most other classification methods used to categorize biosonar sounds in bats first extract individual call parameters, such as call duration and frequency content, from high quality sound recordings. Future experiments might analyze a wide array of echolocation recordings (such as pulse trains recorded from different bat species) to compare SSIMS classification performance against other methods that require more information than timing patterns alone. For example, researchers have used discriminant function analysis (DFA) and artificial neural-network analyses to classify echolocation calls based on individual call parameters (duration, frequency, sweep rate, repetition rate, etc.,) (Parsons and Jones, 2000; Luís et al., 2016). Future work might compare SSIMS classification with DFA.

Expanding the cost function to include other parameters (spectral components, for example) could potentially improve SSIMS classification. Comparing the results obtained using different cost function metrics could then be used to assess the relative importance of different signal properties. SSIMS might also be employed to compare selected segments of longer echolocation pulse sequences. For example, although the current study analyzed flights as a whole, the stereotyped search, approach, and terminal phases of insect hunting (see section “Introduction”) (Griffin et al., 1960) can be delineated and selectively compared using this method. SSIMS may be an especially beneficial tool when applied to field recordings.

There has been particular interest in the terminal buzz emitted by echolocating bats (**Figure 3B**) (Geberl et al., 2015; Hulgard and Ratcliffe, 2016), and pulse-packets or burst-pulses emitted by dolphins (Finneran, 2013; Finneran et al., 2014; Luís et al., 2016). These behaviors are related to prey capture and long-distance target detection, respectively. It would be interesting to test whether SSIMS is predictive of behavioral outcomes, or can identify different species. For example, SSIMS could be used to discriminate terminal buzzes recorded from successful captures with unsuccessful ones, and an estimate of the number of prey eaten could be obtained without the need for video confirmation. In a study with wild echolocating dolphins, researchers found that the repetition rate was a reliable indicator of signal type for call classification analysis (Luís et al., 2016). Repetition rate averages out the fine temporal information that is present in these complex call patterns, and SSIMS may be an even more powerful tool for predicting behaviors of animal recorded vocalizing in the field. Since SSIMS used only the timing information between calls, high-quality recordings of the sound spectrum would not be required for classification purposes. This could lead to an increased ability of field bio-acousticians to characterize even crude sound recordings in the absence of concurrent visual data.

## Author Contributions

AA designed and conducted the experiment. AA and CV-I analyzed the data and wrote the manuscript. JS contributed to the experimental design and execution, and wrote the manuscript.

## Conflict of Interest Statement

The authors declare that the research was conducted in the absence of any commercial or financial relationships that could be construed as a potential conflict of interest.
