# Supplementary material for: Spike Train Similarity Space (SSIMS) Method Detects Effects of Obstacle Proximity and Experience on Temporal Patterning of Bat Biosonar
Source: Front Behav Neurosci. 2018 Feb 8;12:13. doi: 10.3389/fnbeh.2018.00013 (PMC5809465; doi:10.3389/fnbeh.2018.00013)
Supplement: Supplementary file 2 [file Data_Sheet_1.DOCX]

Supplementary Material

Spike-train SIMilarity (SSIMS) method detects effects of obstacle proximity and expectation on temporal patterning of bat biosonarFirst

Alyssa W. Accomando*, Carlos E. Vargas-Irwin, and James A. Simmons

*Correspondence: Alyssa W. Accomando, National Marine Mammal Foundation

2240 Shelter Island Dr., San Diego, CA 92106, USA

Supplementary Data

Supplementary Material should be uploaded separately on submission. Please include any supplementary data, figures and/or tables.

Supplementary material is not typeset so please ensure that all information is clearly presented, the appropriate caption is included in the file and not in the manuscript, and that the style conforms to the rest of the article.

# Supplementary Figures and Tables

## Supplementary Figures


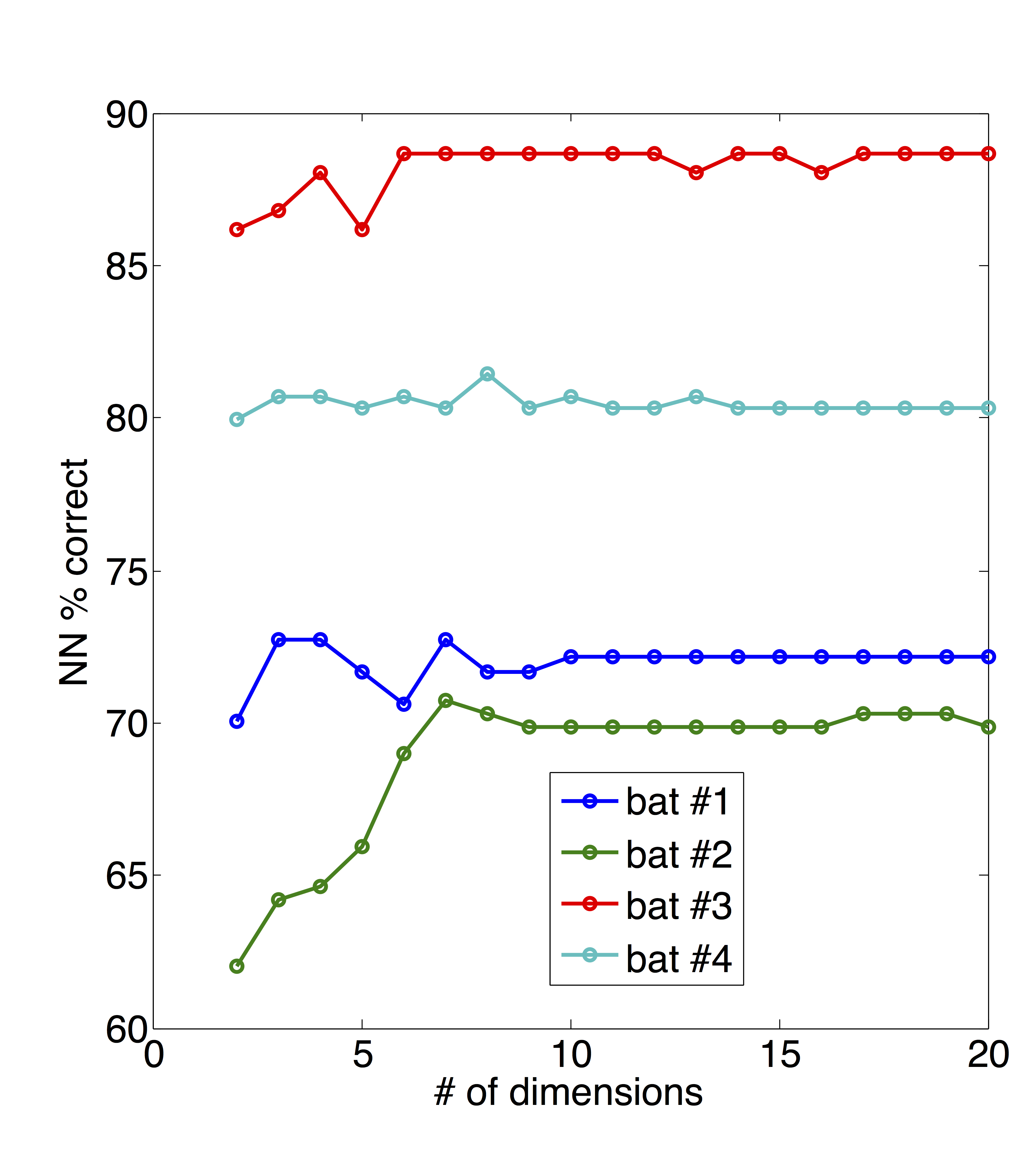


**Supplementary Figure 1.** Dimensionality reduction for SSIMS algorithm implementation. Call patterns for the four individual bats can be separated using low-dimensional representations. Classification performance as a function of similarity space dimensionality is shown. In all cases, asymptotic values are reached between 2 and 6 dimensions. Two-dimensional spaces achieved between 88% and 99% of maximum performance.

**
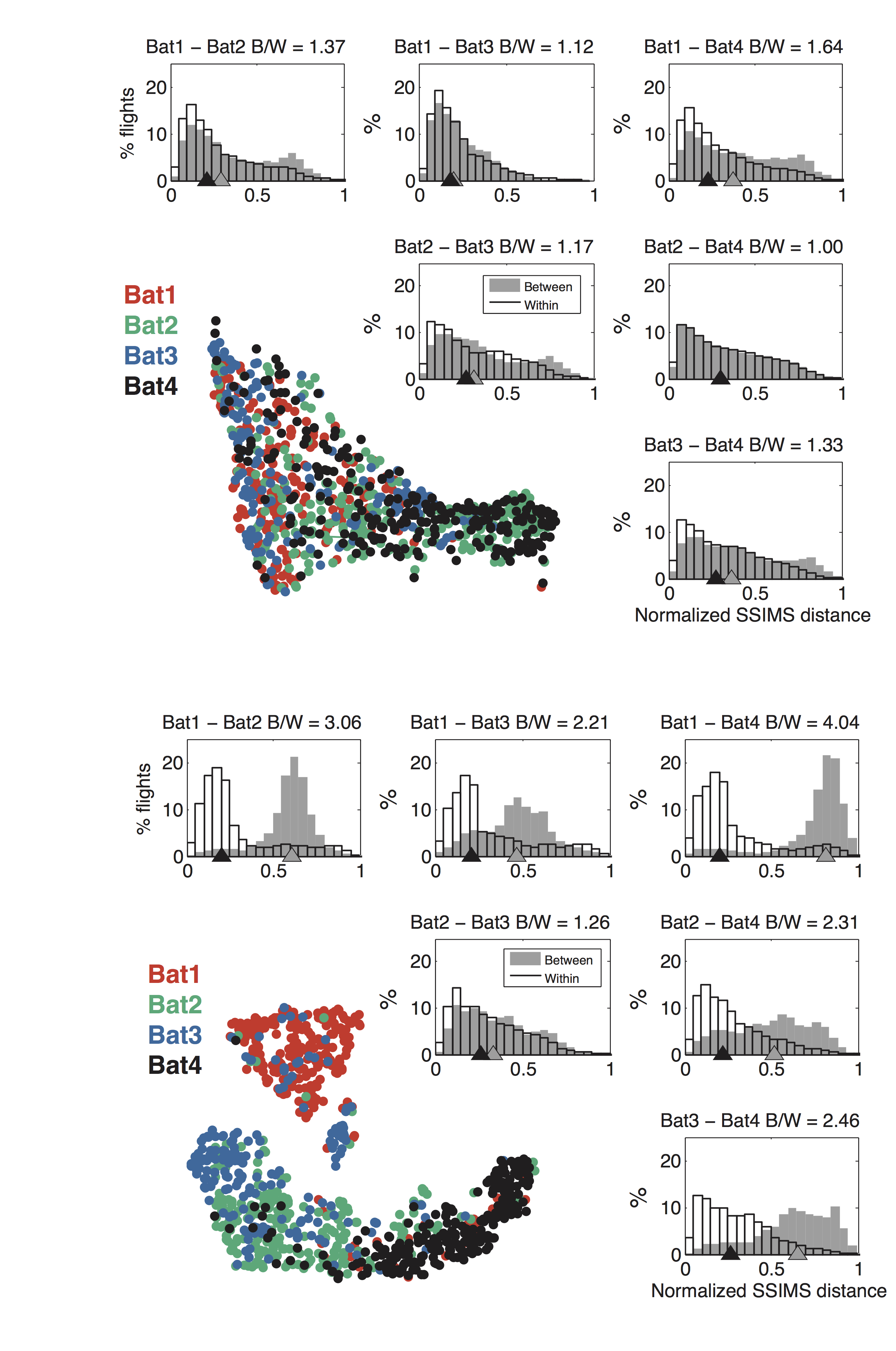
**

**Supplementary Figure 2. Between bat pairwise comparisons.** Flight by flight comparisons between individual bat pairs. Same conventions as Fig 4. All pairwise comparisons of individual bats show significant differences except for Bat 2 and Bat 4 in Experiment 1. Call patterns emitted by Bats 2 and 4 are not statistically different in Experiment 1 (B/W = 1.00), but show separation in Experiment 2 (B/W = 2.31).
